# Hedgehog-Gli1-derived exosomal circ-0011536 mediates peripheral neural remodeling in pancreatic cancer by modulating the miR-451a/VGF axis

**DOI:** 10.1186/s13046-023-02894-9

**Published:** 2023-12-02

**Authors:** Weiqi Dai, Xiaoli Wu, Jingjing Li, Wenxi Tang, Ying Wang, Wenqiang Xu, Dengyu Han, Xiaorong Xu, Xuanfu Xu

**Affiliations:** 1 Department of Gastroenterology, Shidong Hospital of Shanghai, Shidong Hospital Affiliated to University of Shanghai for Science and Technology, No. 999, Shiguang Road, Yangpu District, Shanghai, China; 2 Chongqing General Hospital, Chongqing, China; 3 West China Xiamen Hospital of Sichuan University, Xiamen, Fujian Province China; 4 The People’s Hospital of Pizhou, Xuzhou, Jiangsu Province China; 5 Department of Gastroenterology, Shanghai Tenth People’s Hospital, Tongji University School of Medicine, No. 301, Yanchang Road, Jingan District, Shanghai, China

**Keywords:** Exosome, Circular RNA, Dorsal root ganglion, Pancreatic cancer, Neurological changes

## Abstract

**Background:**

Hedgehog-Gli1 signaling induces development of two common neurological features seen in pancreatic ductal adenocarcinoma (PDAC): peripheral neural invasion (PNI) and peripheral neural remodeling (PNR). However, the underlying molecular mechanisms in cancer cells and nerves within Gli1-derived PNR have not previously been comprehensively analyzed.

**Methods:**

In this study, RNA sequencing was used to screen meaningful circRNAs in PNR. An in vitro model of PNR was subsequently constructed through a co-culture system comprising PDAC cells and murine dorsal root ganglia (DRG) (as the neuronal element), and the relevant mechanisms were explored using a series of molecular biology experiments. A subcutaneous nude mouse tumorigenesis model was established to further verify the occurrence of PNR that was detected in human PDAC samples.

**Results:**

We first confirmed the molecular mechanisms of PNR development through crosstalk between exosomal circ-0011536 and DRG. In Gli1-overpressed PDAC, circ-0011536 is mainly secreted by exosomes. After being ingested by DRG, it can promote the activity of DRG by degrading miR-451a and upregulating the expression of VGF. Overexpression of Gli1 can accelerate the proliferation of subcutaneous tumors in mice and is closely related to the density of nerve plexuses, while downregulating circ-RNA inhibits tumor proliferation and reduces the density of nerve plexuses. In addition, TMA results confirmed that Gli1 overexpression significantly increased the expression of VGF and was closely associated with increased nerve plexus density.

**Conclusion:**

Hedgehog-Gli1-induced exosomal circ-0011536 promoted PNR via the miR-451a/VGF axis, thereby establishing that it may contribute to PDAC-associated nerve changes with activated Hedgehog signaling.

**Supplementary Information:**

The online version contains supplementary material available at 10.1186/s13046-023-02894-9.

## Introduction

Pancreatic cancer is characterized by invasive and non-invasive neurological changes; i.e., pancreatic innervating nerves display perineural invasion (PNI) and neural remodeling (PNR). The incidence of PNI and PNR in pancreatic ductal adenocarcinoma (PDAC) is as high as 100%, which is closely related to the prognosis of pancreatic cancer, and is also an important cause of advanced cancer pain [1, 2]. According to the latest research, neurological changes in tumor cells are not limited to simple diffusion and direct invasion of the nerve fascicle or neuromuscular bundle of the local adjacent nerve, but also involve crosstalk with nerve cells in a variety of ways. These findings have ushered in a new era of neurological research [3].

Zhu et al. confirmed for the first time in 1999 that the expression of nerve growth factor is related to the perineural invasion and pain of pancreatic cancer [4]. It was further confirmed that the expression of nerve growth factor (NGF) receptor in human pancreatic cancer has prognostic value [5, 6]. It is also notable that more than half of PNR cases in pancreatic cancer occur in the extra-pancreatic nerve, far from the tumor. This indicates that the interaction between tumor cells and nerve cells is not just limited to direct adjacent tissue invasion; there is perhaps some carrier responsible for information transmission, and research on exosomes is becoming increasingly popular [7]. The discovery of long-distance transport mechanism of nucleic acid molecules mediated by exosomes is a milestone in tumor microenvironment research, and explains the dilemma of information exchange between cancer cells and distant interstitial cells [8]. The proteins and non-coding RNA (ncRNA) carried by exosomes can be protected from the influence of external proteins and nucleases, making their regulation range more extensive [9, 10]. In PDAC, in particular, there are abundant exosomes involved in information exchange between cells, but the specific nature of their contents and their upregulation and downregulation require further elucidation [11].

Hedgehog (Hh) signaling pathway is one of the basic signaling pathways guiding embryonic development. In the gastrula stage, the disappearance of Sonic Hedgehog expression is a necessary condition for pancreatic organ specialization, and this is overexpressed in pancreatic cancer [12, 13]. Studies have shown that the activation of Hh signaling pathway is positively related to peripheral nerve invasion in pancreatic cancer and neuropathic pain, and may be related to the upregulation of some neurotrophic factors, such as NGF and matrix metalloproteinases [14]. Therefore, the activation of Hh signaling pathway may be one of the important regulatory pathways in the interaction between pancreatic cancer cells and neural cells. NcRNA, owing to its powerful biological functions, has rapidly become a focus of scientific research. Circular RNA (circRNA), which is different from linear RNA, has become a research hotspot in various fields, owing to its stable structure, high conservation, and rich expression [15, 16]. CircRNA is formed through regulation and competition between back splicing and classical splicing. This formation mode substantially differs from the classical splicing mode of long-chain RNA [17]. CircRNA is mainly involved in various biological processes of pancreatic cancer by acting as a miRNA sponge to competitively inhibit mRNA expression, participate in selective splicing or transcription, and other mechanisms [18, 19].

In this study, we seek to explore the role of Gli1-derived exosomal circ-0011536 in the development of neurological changes in pancreatic cancer and investigate the underlying molecular mechanism by regulation of the Hh signaling pathway. Our data further suggest that exosomal circ-0011536 induces neurogenesis, reprogramming, and axon formation of nascent dorsal root ganglion (DRG) neurons. Mechanistically, circ-0011536 contains multiple microRNA binding targets, functioning as a sponge for miR-451a, which in turn promotes expression of VGF. Collectively, our findings provide the foundation for further study on the role of exosomal circRNAs in progression of neurological changes with activated hedgehog-Gli1 signaling.

## Materials and methods

### Cell culture and clinical samples

Human normal pancreatic ductal epithelial cells (HPDE) and the human pancreatic cancer cell lines ASPC1, SW1990, MIAPACA-2, CFPAC-1, and PANC-1 were obtained from the Chinese Academy of Sciences Committee Type Culture Collection cell bank and incubated in high glucose Dulbecco’s Modified Eagle’s Medium (DMEM, Thermo Fisher, USA) supplemented with 10% fetal bovine serum (FBS, Gibco, USA) at 37 °C in 5% CO_2_. A total of 90 patients who underwent operation at Shidong Hospital signed informed consent forms between January 2018 and December 2022. Whole blood samples were collected from patients and separation of plasma exosomes was done, according to the manufacturer’s protocol (4484450, Thermo Fisher, USA). The clinical characteristics of the 90 patients included in the study are shown in Table 1. The pathological criteria for PNR diagnosis were adapted from Liebig et al. [20]. A total of 60 paired and 30 unpaired PDAC specimens from patients were subjected to tissue microarray analysis (TMA). The experimental protocol was conducted in accordance with the Declaration of Helsinki and was approved by the Ethics Committee of Shidong Hospital.

**Table 1:** Baseline data for patients with pancreatic cancer

Characteristic	*N*	Low expression	High expression	*p*
N	90	36	54	
Age				0.829
> 60	50	20	30	
≤ 60	40	16	24	
Sex				0.053
Female	58	28	30	
Male	32	8	24	
TNM stage				0.321
I	58	22	26	
II	42	14	28	
PNR				< 0.001
Negative	56	12	44	
Positive	34	24	10	

### Plasmid and oligonucleotide transfection

For lenti-Gli1 construction, HEK293T was used for virus packaging after the co-transfection of pWPXL-Gli1 with packaging plasmid psPAX2 and envelope plasmid pMD2.G (Addgene, city, state, USA) by Lipofectamine 3000 (Invitrogen, USA). The efficiency of transfection was examined by qPCR and Western blot analysis. The plasmid pcDNA3.1-CMV-circ-0001261 was constructed by GenePharma (Shanghai, China). Small interfering RNAs (siRNAs) against negative control, circ-0001261, and VGF, as well as siRNAs against miR-negative control and miR-451a, were designed by RiboBio (Guangzhou, China). Lipofectamine 3000 (Invitrogen, USA) was used to transfect cells, according to the manufacturer’s instructions. The sequences are depicted in Supplement 1.

### Western blotting

Cells were mixed in a RIPA lysis buffer (Millipore, USA) and protease inhibitor cocktail (Roche, Germany) for 60 min before heating for 10 min at 100℃. The Bicinchoninic Acid (BCA) protein assay (Beyotime, China) was used to test protein concentration. A total of 50 μg protein was probed overnight at 4℃ with the indicated primary antibodies. The antibodies used in the study were as follows: Gli1 (ab217326, Abcam, UK), β-actin (66009–1-Ig, Proteintech, China), Ki-67 (ab16667, Abcam, UK), E-cadherin (60335–1-Ig, Proteintech, China), Caspase-3 (ab32351, Abcam, UK), VGF (26781–1-AP, Proteintech, China), CD63 (67605–1-Ig, Proteintech, China), TSG101 (67381–1-Ig, Proteintech, China), Calnexin (A4846, Abclonal, China), and fluorescence-conjugated secondary antibody (DyLight 800, Odyssey, USA). The Odyssey two-color infrared laser imaging system (LI-COR Biosciences, USA) was used to scan the membrane and assess staining.

### Polymerase Chain Reaction (PCR)

Total RNA was extracted by Trizol reagent (Invitrogen, USA), in accordance with the manufacturer’s instructions. Total RNA (1,000 ng) was used to generate cDNA with SuperScript II reverse transcriptase and Oligo (dT) (Invitrogen, USA). The qRT-PCR experiments were conducted with a real-time PCR kit (Takara, China) and analyzed according to the equation 2^−ΔΔCt^ [ΔCt = Ct-Ct (GAPDH)]. A fold change greater than 1 was defined as high, and a fold change ≤ 1 was defined as low. Primer sequences are shown in Supplement 1.

### Isolation of DRG cells and cell image acquisition

We placed the newborn mice in iced PBS solution under sterile conditions. After decapitation, we inserted a micro-shear from the opening of the cervical spinal canal, removed the exposed spinal cord, carefully extracted the DRG with a fine microscope, and peeled off its capsule. The separated tissue blocks were stored in iced DMEM culture medium, and then collected into a culture dish containing 1 mg/mL collagenase solution for digestion at 37℃ overnight. After adding 10 drops of FBS to stop digestion, we gently blew and centrifuged at 1,000 rpm for 5 min to remove the supernatant. Then, the single cell suspension was inoculated in a 6-well plate for 4 to 6 h. The serum-free culture medium (neurobasal + B27 + glutamine + 20 ng/mL NGF) was used for culture. On the third day of inoculation, the mitotic inhibitor ARA-C was added to inhibit the proliferation of non-neural cells. Representative higher magnification images were taken with an inverted Nikon TE 200, and the total neurite length per neuron was automatically tested using the neurite outgrowth application in MetaMorph (Molecular Devices). Briefly, we captured five representative DRG images from different fields of each culture group and assembled them into a general view in MetaMorph software. Neurite length was automatically recognized, calculated, and exported through the MetaMorph's neural synapse analysis module for later analysis. Recombinant human NGF protein was purchased from Proteintech (HZ-1222, Wuhan, China).

### Cell proliferation, invasion, and chemotaxis

We inoculated the cell suspension in a 96-well plate (approximately 100μL per well). The cells were treated after adhering to the plate. We then added 10μL CCK-8 solution (Dojindo, Japan) to each well, put the culture plate into the incubator for incubation for 2 h. The optical density (OD) at 450 nm wavelength was measured with a microplate reader.

We diluted the Matrigel with serum-free cold cell culture medium DMEM and added it to the upper chamber of 24-well Transwell. We incubated a Transwell insert (Thermo Fisher, USA) at 37℃ for at least 4 to 5 h. The obtained cells were washed with culture medium, and 200μL of cell suspension was added into the upper chamber at a density of 5 × 10^5^ cells/mL. Subsequently, 600μL of cell culture medium was added to the lower chamber before incubation. After 24 h of culture, the cells remaining on the membrane were stained with 0.1% crystal violet for counting and analysis. In the chemotaxis experiment, PDAC and DRG cells were also added into the upper and lower chambers of Transwell at 1:1 ratio, and crystal violet showed the proportion of transmembrane penetrating cells.

### Annexin-V/PI staining

DRG cells were incubated in six-well plates and treated as indicated. After treatment at room temperature for 15 min in Annexin-V/PI (BD Biosciences, USA), the cells were tested and analyzed by flow cytometry (BD FACSCanto, USA).

### Exosome isolation and identification

Exosomal fraction from 30 mL of cell culture supernatant was extracted by ExoQuick™ Exosome Precipitation Solution, according to the manufacturer’s recommendations (System Biosciences, USA). Briefly, 1/2 volume of ExoQuick Solution was added to cell culture supernatant and samples were refrigerated at 4 °C overnight. The exosome was isolated by centrifugation at 10,000 g for 60 min. The isolated exosome was detected by Grainsize Analyzer (Zetasizer Nano ZS, UK). The measuring range of the zetasizer is from approximately 0.1 nm to 10 µm. The sizes and aggregational states of exosome were further examined using a TEM (JEM-2100, Japan).

For uptake experiments, purified exosomes were labeled using the PKH26 labeling kit (Sigma Aldrich, USA), according to the manufacturer’s instructions. In brief, exosomes were mixed in solvent C (containing PKH26 dye) and incubated for about 5 min. The labeled exosomes were then co-cultured with DRG cells for 24 h. After slides were stained with 4ʹ,6-diamidino-2-phenylindole for 10 min, they were visualized under a confocal laser scanning microscope.

### RNA sequencing

The total RNA extracted from DRG and PDAC cells was depleted of linear RNA and ribosomal RNA. RNA was then fragmented and reverse transcribed. After linking to the sequencing adaptor, a library was obtained for the whole genome sequencing. The raw sequencing reads were mapped to the reference human genome through TopHat2 and TopHat-Fusion sequentially. Mapped reads (from TopHat2) and back-spliced reads (from TopHat-Fusion) were used to quantify the enrichment of each circRNAs candidate, indicated in RPM (reads per million mapped reads). The expression level of each circRNA was compared by DEG seq and the images were drawn by Sangerbox [21].

### Luciferase reporter assay

Luciferase reporter constructs containing circ-0001261-miR-451a or VGF mRNA 3ʹUTR-miR-451a binding sequences (circ-0001261-WT or VGF-WT) or mutant sequences (circ-0001261-MUT or VGF-MUT) and miR-451a mimic or miR-NC were co-transfected with psiCHECK-2 dual-luciferase plasmid as well as the Renilla luciferase gene. Before luciferase reporter assay, DRG cells (1 × 10^5^ cells/well) were seeded in 24-well plates for 24 h. After 48 h transfection, the cellular luciferase activity was examined by a luciferase reporter assay system (Promega, USA), in accordance with the manufacturer’s protocol.

### In vivo study

Nude mice (males, 4 weeks old) were subcutaneously injected with lenti-Gli1 CFPAC-1 or CFPAC-1 cells (1 × 10^7^cells/mouse) to form PDAC. With regard to the si-circRNA treatment, 1 × 10^8^ plaque-forming units/100 mL lentivirus vectors of si-circ-0001261 were transduced into nude mice through tail injection. All animal experimental procedures were approved by the Institutional Animal Use Committee of Shidong Hospital. The tumor volume was measured every three days and the mice were killed three weeks after injection for further analysis of tumor tissues. The number of intra-PDAC nerves was analyzed by calculation of the average number of PGP9.5^+^ cells from five randomly selected PGP9.5 immunostaining fields per culture group.

### Immunohistochemistry and immunofluorescence

For immunohistochemistry experiments, we used negative control (tissue without primary antibody) and positive control (human breast carcinoma) tissues. For immunofluorescence experiments, human peripheral nerve sheath tumor tissue (PNST) from Shidong Hospital was used as a representative Gli1 and PGP9.5 immunostaining positive control. The tumor tissue was embedded in paraffin, and the other part was dehydrated with sucrose and then embedded with optimal cutting temperature compound (OCT), and subsequently sliced for standby. After the paraffin section was dewaxed with xylene and dehydrated with alcohol, 3% H_2_O_2_ was added to remove endogenous catalase. After adding serum to block the reaction, we added primary and second antibodies, and finally used diaminobenzidine (DAB) to develop color and obtain images under the microscope. Tissue sections were incubated with rabbit monoclonal anti-Ki-67 (1:150; ab16667; Abcam), rabbit polyclonal anti-VGF (1:100; 26781–1-AP; Proteintech), rabbit polyclonal anti-S100B (1:400;15146–1-AP; Proteintech), mouse monoclonal anti-Gli1 (1:400; 66905–1-Ig; Proteintech), or anti-PGP9.5 (1:100; ab8189; Abcam). For quantification, blinded semi-quantitative scoring was used, and the results were quantified as negative (< 5%) and positive (≥ 5%).

### Statistical analysis

All data are expressed as the mean ± standard deviation (SD) and were analyzed by SPSS 22.0 software (IBM, USA). Correlations between clinical categorical parameters and circRNA levels were evaluated by χ2 test. One-way analysis of variance (ANOVA) was used to compare differences among three groups. The student’s t-test was employed to compare grouped differences when the data followed a normal distribution and the Mann–Whitney test was recommended for the comparison of two independent groups. In all cases, a *P*-value < 0.05 was considered to be statistically significant.

## Results

### Gli1 can promote the proliferation and invasion of PDAC cells

**Fig. 1:**
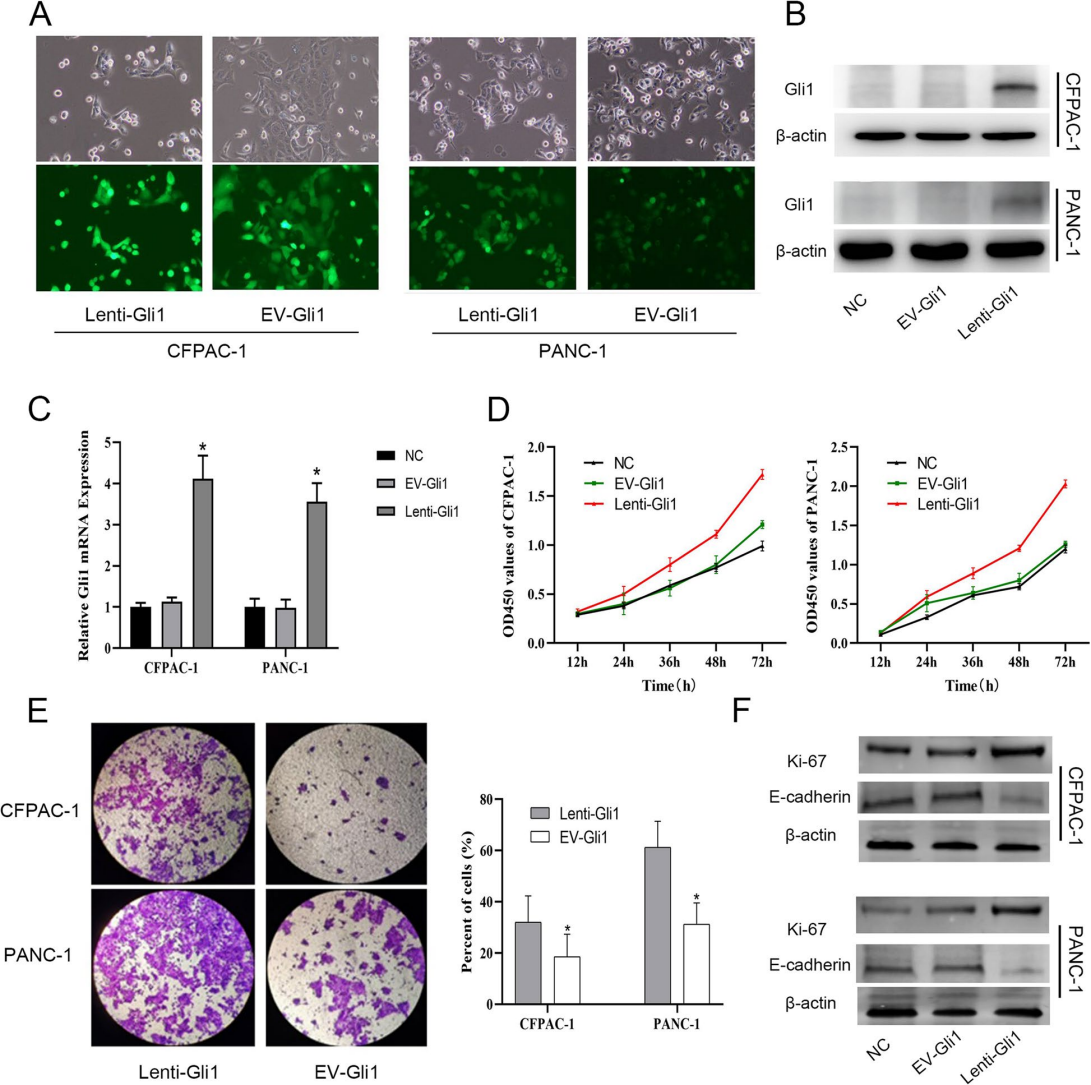
The effect of Gli1 in PDAC cells. **A** Fluorescence expression of lentivirus transfection (200 ×). **B** Protein levels of Gli1 were measured by Western blotting. **C** qRT-PCR was used to assess relative expression levels of Gli1. **D** OD450 values were measured with a CCK-8 kit. **E** The number of invasive cells is shown by crystal violet staining. **F** Protein levels of Ki-67, E-cadherin, and Vimentin were measured by Western blotting. *P < 0.05 for lenti-Gli1 vs. EV-Gli1

To investigate whether Gli1 upregulation promotes tumor progression of PDAC cell lines, we first examined Gli1 expression by qRT-PCR and Western blotting in five PDAC cell lines. At least four PDAC cell lines (80.0%) showed upregulation of Gli1 expression with respect to that in HPDE, whereas both the mRNA and protein expression of Gli1 was relatively low in CFPAC and PANC-1 cells (Supplementary Fig. 1A and B). To further examine the functional roles of Gli1 in PDAC cell progression, we constructed Gli1 lentivirus (lenti-Gli1) in CFPAC and PANC-1 cells, and used empty virus as a control (EV-Gli1). Cell fluorescence results under microscope showed that the transfection effect was more than 80%. The effect of overexpression of Gli1 at RNA and protein levels was verified by qPCR and Westernblotting (Fig. 1A–C). Furthermore, we used CCK-8 method to detect the effect of upregulation of Gli1 expression on the proliferation of PDAC cells. The results showed that the cell proliferation rate of the overexpression Gli1 group was significantly higher than that of the empty group (Fig. 1D). Transwell method was used to detect the role of Gli1 in the invasion ability of PDAC cells. The results showed that the number of cells passing through the bottom polycarbonate membrane in lenti-Gli1 group was significantly higher than that in the EV-Gli1 group after 24 h of culture, and the difference in cell count was statistically significant (Fig. 1E). Similarly, Western blot results confirmed that the genes Ki-67 and E-cadherin, related to proliferation and invasion, also showed a trend consistent with the function. E-cadherin in the lenti-Gli1 group decreased, while Ki-67 was upregulated, indicating that overexpression of Gli1 effectively improved the proliferation and invasion ability of PDAC cells (Fig. 1F). Moreover, the protein level of E-cadherin was diminished in Lenti-Gli1 cells, and showed an inverse relationship with Vimentin levels, thus suggesting that Gli1 overexpression might induce epithelial mesenchymal transition (EMT) in PDAC cells (Fig. 1F).

### Gli1 can induce DRG chemotaxis mediated by PDAC cells

**Fig. 2:**
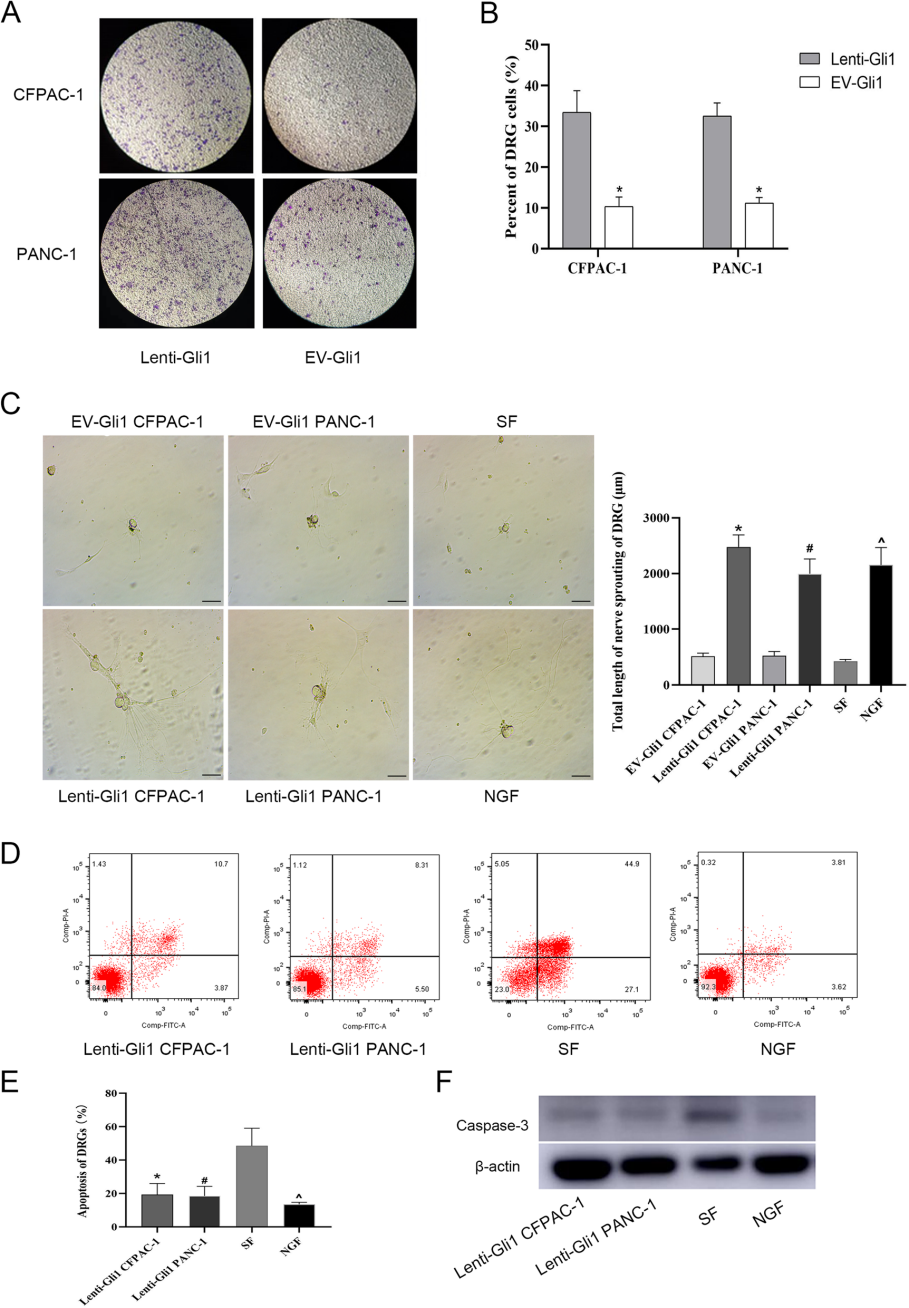
The effect of Gli1-overexpressing PDAC cell supernatant on DRG. **A**, **B** The number of chemotactic DRG cells in PDACs. *P < 0.05 for lenti-Gli1 vs. EV-Gli1. **C** The axon length of DRG (200 ×) and statistical analysis. **D**, **E** Apoptosis of DRG was determined by flow cytometry. **F** Protein levels of caspase-3 were measured by Western blotting. *^#^*P* < 0.05 for lenti-Gli1 vs. SF; ^*P* < 0.05 for NGF vs. SF

To further facilitate characterization of the underlying molecular basis for the observed neuronal changes, we constructed an in vitro co-culture model, using DRG cells as the neuronal element. The DRG of mice were extracted, and the PDAC-DRG co-culture model was constructed (DRGs for small cell and PDACs for lower cell). The experimental results showed that compared with the EV-Gli1 control group, the number of DRG cells passing through the Transwell cell in the lenti-Gli1 group was significantly increased (Fig. 2A, B). In order to further study the functional role of Gli1 signal in neuronal plasticity, we used NGF as a control to observe the axonal extension length of DRG. The results suggested that the lenti-Gli1 group promoted axon extension in DRG in PDAC cells, which was similar to the effect of NGF production. The empty serum without NGF (SF group) was used as the control (Fig. 2C). In addition, Gli1 overexpression also protected DRG from apoptosis induced by serum starvation. As measured by Annexin V/PI, when the Gli1 overexpression PDAC cells were co-cultured with DRG, the expression of caspase-3 in the cells was significantly reduced (Fig. 2D–F). The above studies showed that the activation of Gli1 in PDAC cells can promote the chemotaxis of DRG cells and axonal nerve extension, while avoiding the occurrence of starvation-induced apoptosis.

### Gli1 acts on DRG through PDAC-induced exosomes

**Fig. 3:**
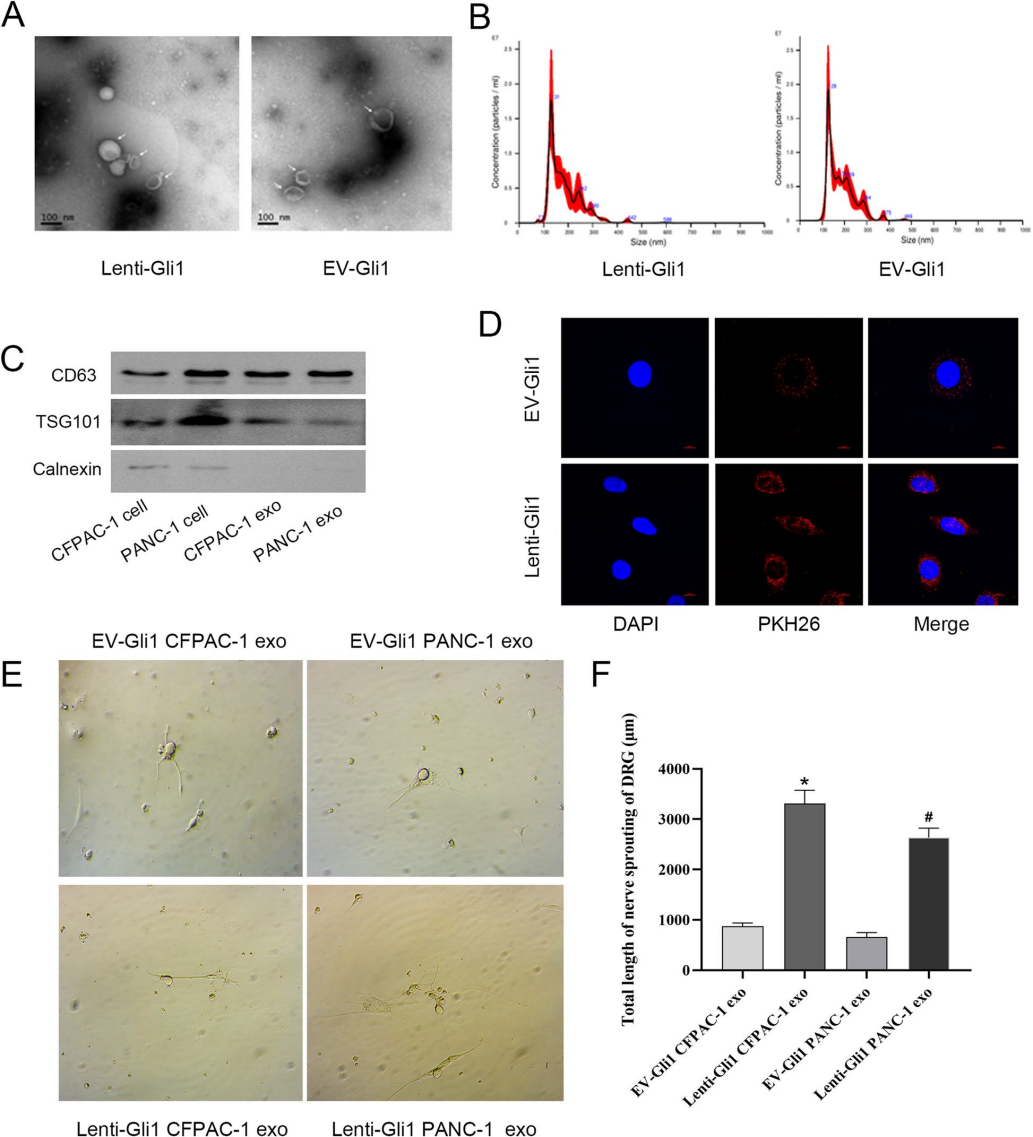
The identification of exosomes and their effects on DRG. **A**, **B** Exosomes were identified by TEM (30000 ×) and NTA. **C** Protein levels of CD63, TSG101, and Calnexin were measured by Western blotting. **D** Exosome uptake was detected by confocal detection (400 ×). **E**, **F** The axon length of DRG (200 ×) and statistical analysis. *^#^*P* < 0.05 for lenti-Gli1 vs. EV-Gli1

We extracted the supernatant for exosome separation, and verified the substances obtained by transmission electron microscopy, nano particle tracking analysis (NTA) analysis, and Western blotting test. The results of electron microscopy showed that the shape of the extract was transparent like a saucer, with a complete vesicle membrane structure. The results of NTA showed that the volume of exosomes extracted from the supernatant of the two groups of cells was mainly 130 nm, which was consistent with the previous literature, verifying the existence of exosomes in the pancreatic cancer cell line CFPAC-1. The Western blotting results showed that the exosome marker proteins CD63 and TSG101 were positive, while the negative control Calnexin was positive in the cell line, but not in the exosomes (Fig. 3A–C). In order to confirm whether the exosomes derived from PDAC cells can be transferred to the DRG receptor, we extracted the exosomes of the lenti-Gli1 and EV-Gli1 groups, labeled them with PKH26 dye, filtered the exosomes, and added them into the DRG, co-cultured with the DRG for 24 h, and showed that the exosomes were focused around the cells and absorbed by them through confocal display (Fig. 3D). At the same time, the exosome of the lenti-Gli1 group lengthened the axon extension of DRG (Fig. 3E, F). This suggests that the effect on DRG may be realized by the exosomes originating from PDAC cells.

### Screening and verification of circ-RNA in exosomes carrying transmission signals

**Fig. 4:**
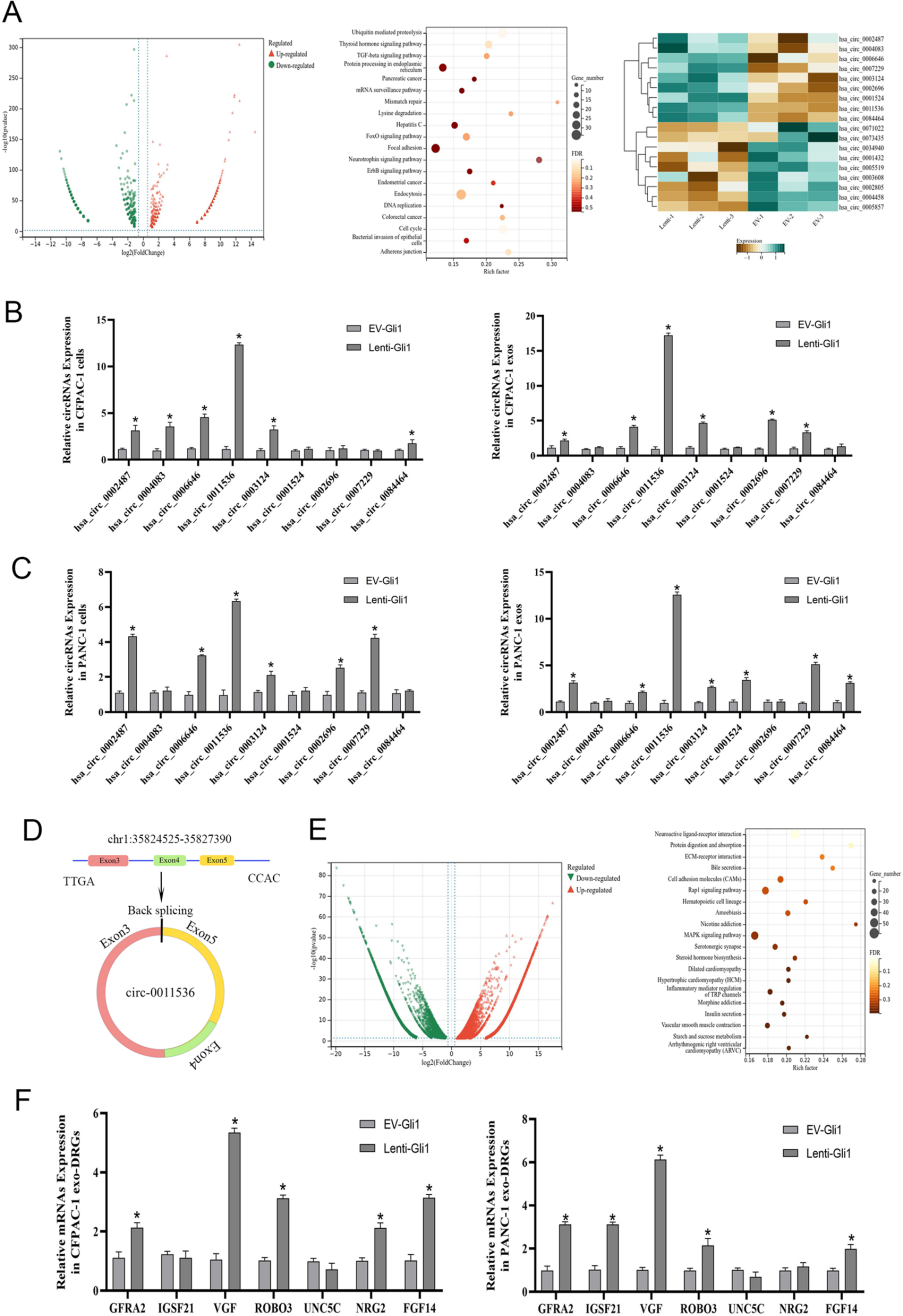
The identification of has-circ-0011536 in exosomes and VGF in DRGs. **A** The volcano plot and clustered heatmap compare the expression of circRNAs for lenti-Gli1 and EV-Gli1. KEGG analysis of DEGs. The circRNAs were classified according to the Pearson correlation. **B**, **C** qRT-PCR was used to assess relative expression levels of circRNAs in cells and exosomes. **D** Schematic illustration of circPRMT5 locus with specific primers. **E** Volcano plot compares the expression of mRNAs for lenti-Gli1 and EV-Gli1-induced DRG cells. KEGG analysis of DEGs. **F** qRT-PCR was used to assess relative expression levels of mRNAs in DRG cells. *P < 0.05 for lenti-Gli1 vs. EV-Gli1 (by Sangerbox [21])

We extracted RNA and performed RNA-seq on CFPAC-1 cells overexpressing Gli1; an empty virus group served as a control. Among the identified lncRNAs, circRNAs, and microRNAs, circRNAs were significantly different. We identified 2,621 differentially expressed genes on the basis of log_2_(fold changes) > 1, *P* < 0.05, and FDR < 0.05, of which 1,530 were upregulated, and 1,091 were downregulated. KEGG pathway enrichment analysis was performed on the differential genes, and a total of 18 circRNAs associated withthe Neurotropin signaling pathway were screened, including nine upregulated and nine downregulated genes (Fig. 4A). To elucidate the underlying mechanism, we assessed the expression levels of the nine upregulated genes (*P* < 0.001) with the most significant differences in cells and exosomes. The expression of hsa-circ-0011536 increased synchronously, and the difference was significant (Fig. 4B–D). Similarly, we treated DRG with exosomes extracted from cells with overexpression of Gli1 and performed sequencing. A total of 6,718 upregulated genes and 5,578 downregulated genes were identified. After clustering, 59 genes were found to be involved in the Neuroactive ligand-receptor interaction pathway (Fig. 4E). The first seven significantly different genes were selected for qPCR detection in DRG total RNA. VGF was found to be upregulated in lenti-Gli1-treated DRG cells (Fig. 4F). These findings suggested that exosomes might carry has-circ-0011536 and transmit it to DRG cells, thereby altering VGF and leading to downstream effects.

### Exosomes carrying circ-0011536 act on DRG cells through miR-451a

**Fig. 5:**
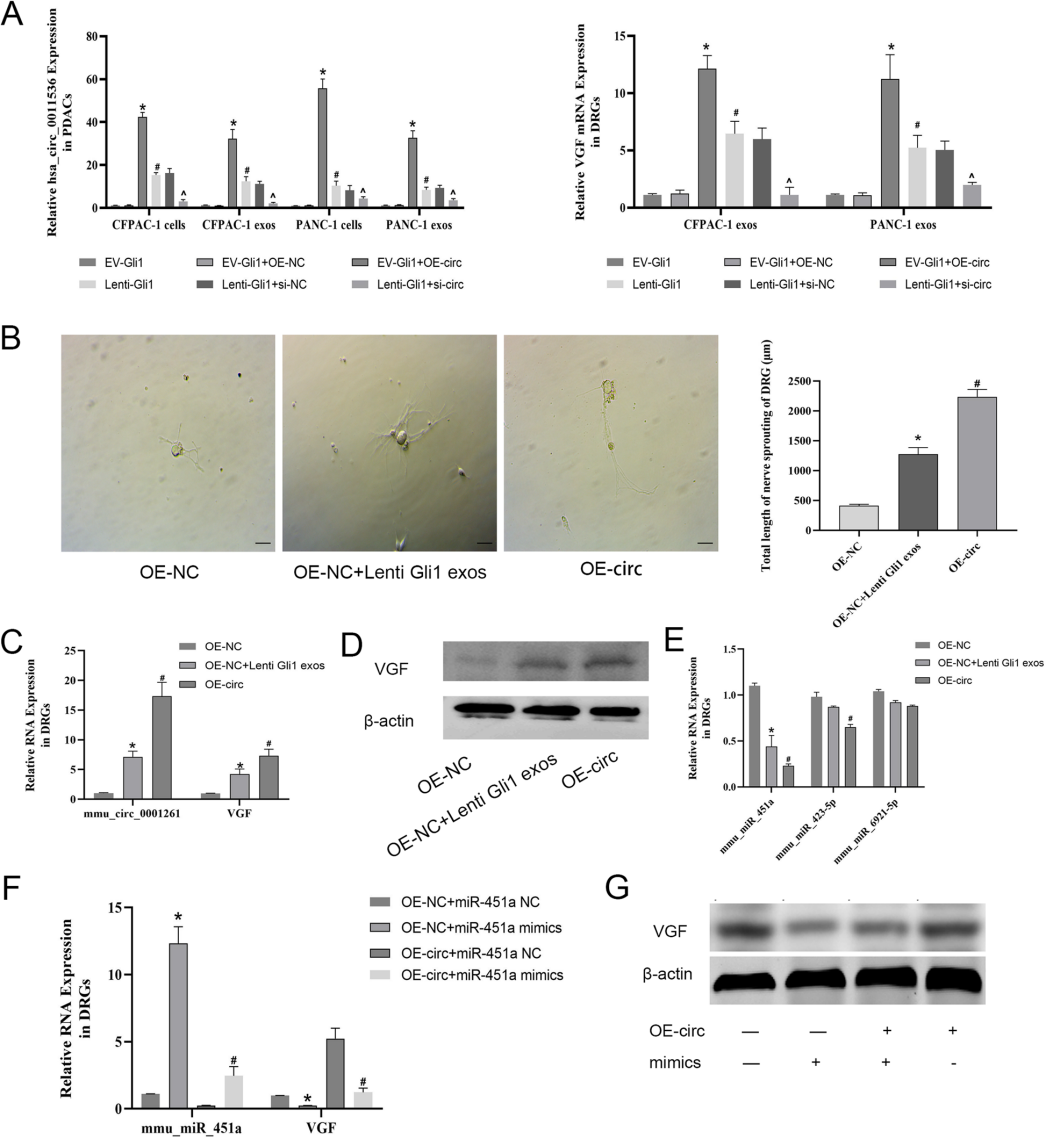
The screening and identification of miR-451a in DRG cells. **A** qRT-PCR was used to assess relative expression levels of has-circ-0011536 and VGF. *P < 0.05 for EV-Gli1 + OE-circ vs. EV-Gli1 + OE-NC; ^#^*P* < 0.05 for lenti-Gli1 vs. EV-Gli1; ^^^*P* < 0.05 for lenti-Gli1 + si-NC vs. lenti-Gli1 + si-circ. **B** The axon length of DRG (200 ×). **C** qRT-PCR was used to assess relative expression levels of mmu-circ-0001261 and VGF. **D**, **G** Protein levels of VGF were measured by Western blotting. **E** qRT-PCR was used to assess relative expression levels of miRNAs and VGF. *P < 0.05 for OE-NC + lenti Gli1 exosomes vs. OE-NC; ^#^*P* < 0.05 for OE-circ vs. OE-NC. **F** qRT-PCR was used to assess relative expression levels of mmu-miR-451a and VGF. *P < 0.05 for OE-NC + miR-451a mimics vs. OE-NC + miR-451a NC; ^#^*P* < 0.05 for OE-circ + miR-451a mimics vs. OE-circ + miR-451a NC

We constructed vectors for overexpression and knockdown of hsa-circ-0011536 in EV-Gli1 and lenti-Gli1 PDAC cells, respectively. After verifying the circRNA expression, we preliminarily assessed the expression of VGF in DRG cells showing an exocrine effect. The expression of VGF was consistent with that of the circRNA change (Fig. 5A). In addition, the exosomes secreted by lenti-has-circ-0011536 and lenti-Gli1 CFPAC-1 cells similarly promoted axon elongation (Fig. 5B). Because DRG and PDAC cells come from different species and genera, has-circ-0011536 was converted into the homologous gene mmu-circ-0001261 in DRG cells for detection. Sequence comparison analysis indicated that both genes were highly conserved. We subsequently detected VGF mRNA and protein in DRG cells treated with exosomes. Secretions from CFPAC-1 cells with overexpression of Gli1 and has-circ-0011536 showed elevated expression of mmu-cic-0001261 and VGF (Fig. 5C, D). We used luciferase assays to assess binding of mmu-cic-0001261 to VGF; the results did not demonstrate direct binding, thereby indicating that mmu-cic-0001261 might be involved in the regulation of endogenous competitive RNA. The ENCORI and Circineracotome databases, predicted that 15 miRNAs may be complementary to mmu-cic-0001261, and 33 miRNAs upstream of VGF were predicted by ENCORI, TargetScan, miRTarBase, and miRDB, and three common miRNAs crossed by the two (mmu-miR-423-5p, mmu-miR-451a, mmu-miR-6921-5P) were found to be highly conserved and significantly different after DRG validation (Fig. 5E). To further verify the effects of miRNAs, we constructed a DRG cell model with overexpression of mmu-cic-0001261 to simulate the effects of lenti-Gli1 exosomes. After addition of miR-451a mimics, the mRNA and protein of VGF were downregulated (Fig. 5F, G). According to the above results, we preliminarily determined that PDAC cells act on and mediate neural cell change through a Gli1-circ-0011536-miR-451a-VGF pathway.

### Gli1-derived exosomal circ-0011536 mediates PNR through the miR-451a/VGF axis

**Fig. 6:**
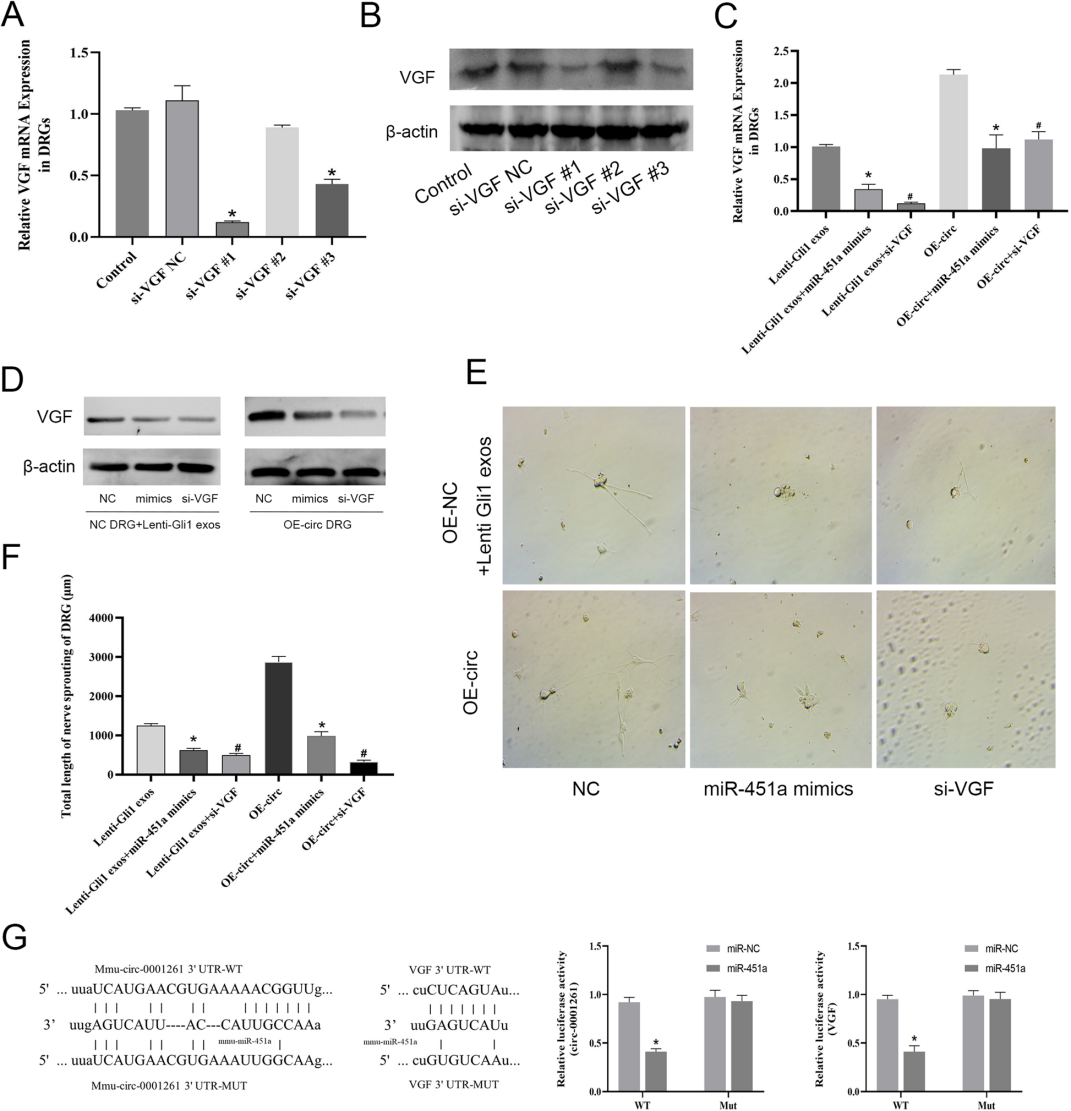
The verification of mechanism of exosomal circ-0011536 as ceRNA and competitive binding of miRNA-451a. **A** qRT-PCR was used to assess relative expression levels of VGF. *P < 0.05 for si-VGF vs. si-VGF NC. **B**, **D** Protein levels of VGF were measured by Western blotting. **C** qRT-PCR was used to assess relative expression levels of VGF. **E**, **F** The axon length of DRG (200 ×). *P < 0.05 for lenti-Gli1 exos/OE-circ + miR-451a mimics vs. lenti-Gli1 exosomes; ^#^*P* < 0.05 for lenti-Gli1 exosomes/OE-circ + si-VGF vs. lenti-Gli1 exosomes. **G** Sequences of wild-type and mutant mmu-circ-0001261 and VGF that disrupt interaction with miR-451a are shown. The relative dual luciferase activity isindicated. *P < 0.05 for miR-451a vs. miR-NC

We constructed VGF siRNA and verified silencing in DRG cells, to determine the most efficient siRNA (Fig. 6A, B). Subsequently, we simulated the DRG cell model after the interaction of lenti-Gli1 exosomes with the DRG cells overexpressing mmu-cic-0001261, and added miR-451a mimics and si-VGF for rescue experiments. As expected, the overexpression of miR-451a or the silencing of VGF decreased the mRNA and protein expression of VGF, and also affected the axon extension of DRGs (Fig. 6C–F). Luciferase reporter assays indicated that mmu-cic-0001261 and miR-451a simulated therapy inhibited WT signal, whereas mmu-cic-0001261 mutation eliminated the inhibition of miR-451a mimics. In addition, miR-451a mimics decreased the VGF-WT luciferase reporter signal in DRG cells but had no effect on the VGF-MUT vector (Fig. 6G). Our data demonstrated that in vitro, in Gli1-overexpressing PDAC cells, has-circ-0011536 carried by exosomes regulates the neuronal changes in DRG cells via the miR-451a/VGF axis.

### Levels of circ-0011536 in plasma exosomes are a prognostic biomarker associated with PNR in PDAC in mice and humans

**Fig. 7:**
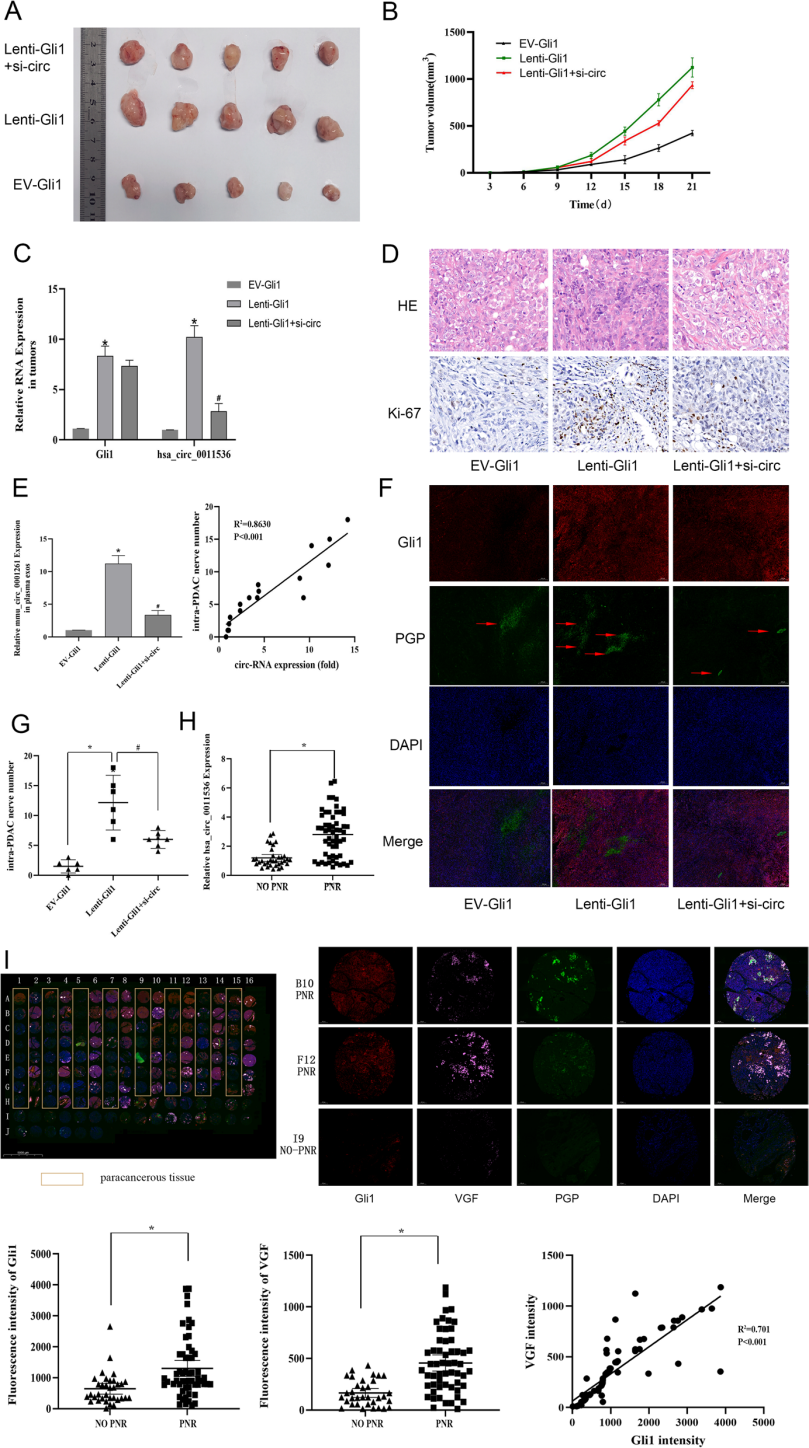
Relationship between exosomes and nerve alterations in vivo. **A** Gross observation of xenograft tumors in nude mice. **B** Changes in tumor volume were recorded at the time points indicated. **C** qRT-PCR was used to assess relative expression levels of Gli1 and has-circ-0011536. **D** HE (200 ×) and immunohistochemical staining (400 ×) of tumors shows the levels of Ki-67. Negative control (NC; tissue without primary antibody) and positive control (PC; human breast carcinoma) was used. **E** Quantification of Ki-67^+^cells in randomly selected areas of the indicated groups (NC: negative control; PC: positive control). Expression of Ki-67 was quantified as a percentage of Ki-67 positive cells with respect to the total cells from randomly selected areas (*n* = 5) in each group. Results are means ± SEM. **P < 0.01 for lenti-Gli1 vs. EV-Gli1; ^##^*P* < 0.01 for lenti-Gli1 + si-circ vs. lenti-Gli1. **F** qRT-PCR was used to assess relative expression levels of mmu-circ-0001261. The correlation between circ-RNA expression and nerve number. **G** Immunofluorescence staining (40 ×) of tumors indicates the levels of Gli1 and PGP 9.5. Representative Gli1 and PGP9.5 immunostaining in positive control (peripheral nerve sheath tumor specimen). **H** The number of intra-PDAC nerves was calculated in tissues. *P < 0.05 for lenti-Gli1 vs. EV-Gli1; ^#^*P* < 0.05 for lenti-Gli1 + si-circ vs. lenti-Gli1. **I** The expression of has-circ-0011536 in exosomes from pancreatic cancer with and without PNR. **J** Immunofluorescence staining for Gli1, PGP9.5, and VGF on a tissue microarray analysis (TMA) slide containing PDACs. *P < 0.05 for PNR vs. NO PNR

To determine the role of circ-0011536 in vivo, we used EV-Gli1 and lenti-Gli1 CFPAC-1 to establish a mouse subcutaneous tumorigenic model. We then conducted circ-siRNA treatment in lenti-Gli1 mice. First, we evaluated proliferation levels with hematoxylin–eosin and Ki-67 staining, Ki-67 nuclear staining was higher in the lenti-Gli1 group and the positive control than in the negative control or EV-Gli1 tissues. However, a significant decrease in Ki-67 expression was observed in lenti-Gli1 tissues after circ-siRNA treatment, thus indicating anti-proliferative action in lenti-Gli1 mice (Fig. 7D, E). Additionally, tumors with Gli1 overexpression grew faster, whereas tumor growth was inhibited after circ-siRNA treatment (Fig. 7A, B).We subsequently detected Gli1 and has-circ-0011536 expression in tumors by PCR, and found that the expression of Gli1 and circ were consistent with the regulatory expectations (Fig. 7C). We extracted exosomes from mouse plasma for detection of mmu-circ-0001261, and measured the number of intra-PDAC nerves by counting the average number of PGP9.5^+^ cells from five randomly selected PGP9.5 immunostaining fields per group. The expression of mmu-circ-0001261 positively correlated with nerve density in the tumors (R^2^ = 0.8630, *P* < 0.001). Mice treated with siRNA showed significant nerve attenuation in tumors (Fig. 7F). In addition, co-immunostaining experiments indicated that PGP9.5 and S100B were highly co-expressed in mice PDAC tissues (Supplementary Fig. 2), thus further suggesting that PGP9.5 can be used for nerve tissue detection in PDAC samples. Moreover, whereas cytoplasmic staining of Gli1 and PGP9.5 was elevated in both the positive control group (PNST specimen) and lenti-Gli1 group, little staining was observed in lenti-Gli1 tissues after circ-siRNA treatment (Fig. 7G). The results of double immunofluorescence staining suggested that the nerve plexus density increased after Gli1 overexpression, but decreased with downregulation of circ-siRNA (Fig. 7G, H). We collected samples from 90 patients with PDAC for analysis. The expression of plasma exosomal circ-0011536 was not associated with age or tumor stage, but was closely associated with PNR (Table 1). Moreover, the expression of has-circ-0011536 in the plasma exosomes of patients with PNR was significantly elevated (Fig. 7I). Finally, we validated the relationship between Gli1 and PNR through TMA using fluorescence triple staining (Fig. 7J). The results confirmed that Gli1 expression was closely associated with PNR and significantly increased expression of VGF (*R*^2^ = 0.7010, *P* < 0.001). The above results suggested that circ-0011536 may be upregulated by Gli1 in tumors and subsequently directly promote an increase in intra-tumoral nerve density, thus serving as a biomarker to predict PNR in pancreatic cancer.

## Discussion

Patients with pancreatic cancer have very poor prognosis and quality of life of. Although surgical treatment is partially effective, the postoperative recurrence rate is very high, and late cancer pain is an important problem. Therefore, the study of neural alterations in pancreatic cancer may enable more accurate removal of lesions with nerve invasion, decrease postoperative recurrence, improve patient quality of life, and aid in prognostic ation of pancreatic cancer and the treatment of patients with cancer pain [22].

Hh signaling, which regulates body mode and organ development during embryogenesis, is static in normal adults, and is closely associated with the occurrence and development of cancer after being activated. Hh pathway inhibitors have been preliminarily applied in clinical practice and have shown good results [13]. The effects of the Hh signaling pathway are achieved through the transcription of downstream target genes upregulated by the nuclear transcription factor Gli1. The effects of Gli1 in promoting pancreatic cancer proliferation and invasion were verified in this study, and our results were consistent with those reported in the literature [23, 24].

In a previous study, Dai et al. established a co-culture model of pancreatic tumor cells and DRG cells. The authors confirmed that the presence of pancreatic cancer cells stimulates the growth of nerve axons, and observed substantial proliferation of MIA pancreatic cancer cells co-cultured with DRG [25]. Jurcak et al. have further suggested that axon guidance molecules promote perineural invasion and metastasis of orthotopic pancreatic tumors in mice [26]. To construct a reproducible in vitro model of PNR, we incubated DRG cells with lenti-Gli1 PDAC cells. We demonstrated that NGF induced nerve cell survival and neuritogenesis in DRG cells, whereas serum starvation prevented these processes. In addition, PDAC cells co-cultured with DRG cells recapitulated the major phenotypic characteristics of neuronal plasticity observed in tissues. These data provided a proof of principle of the utility of our in vitro model. However, the above studies were limited to the establishment and evaluation of the co-culture model, and information exchange channels require further exploration. New evidence increasing indicates that exosomes are involved in information exchange in pancreas-related diseases, and have close communication or cross-talk with cancer-associated fibroblasts, pancreatic stellate cells, hepatic stellate cells, macrophages, and endothelial cells [27, 28]. In our study, we identified the features of exosomes in both PDAC cell culture media and patient-serum through NTA and TEM. Exosomes from the supernatant of PDAC cells also altered axon length and modulated phenotypic changes after being added to DRG cells. However, the underlying molecular mechanisms require further exploration.

Several studies have shown that exosomes secreted by pancreatic cancer cells are rich in ncRNAs and proteins that mediate the functions of other cells through a variety of mechanisms [29, 30]. Therefore, identifying genes that are regulated by Gli1 and can be carried by exosomes is critical to influence the phenotypic phase of secretory neurons. In the current study, we performed RNA sequencing of Gli1-overpressing PDAC cells in an in vitro model. The most significant differences in circRNAs were analyzed with KEGG cluster analysis, and circRNAs associated with the neurotropin signaling pathway were screened. After a series of validation experiments, we determined that circ-0011536 was highly expressed in cells, and exosomes from cells and patient sera. Functionally, the upregulation of exosomal circ-0011536 potentiated the effects of exosomes on neuronal cell survival and axonal sprouting in DRG cells, thus suggesting that circ-0011536 may contribute to cellular communication through the delivery of exosomes. Similarly, DRG cells treated with exosomes were sequenced to determine the target genes affecting the DRG phenotype. As expected, most genes clustered in the neuroactive ligand-receptor interaction pathway. The significantly different gene VGF was identified by qRT-PCR validation of DRG cells, and was further established to be closely associated with the expression of circ-0011536. In the above experiments, we verified that exosomes serve as a bridge for intercellular communication. PDAC cells transmit Gli1-circ-0011536 signals through exosomes, thereby affecting the shape and function of DRG cells. However, the specific mechanism in DRG requires further exploration. PDAC cells and DRG cells come from different strains, and hsa-circ-0011536’s homolog is the highly conserved mmu-circ-0001261 in mice. We also verified, in a preliminary experiment, that mmu-cic-0001261 did not directly bind VGF; therefore, we speculated that circRNAs might act as an endogenous miRNA sponge, thereby competitively suppressing miRNAs regulation of downstream target genes at the post-transcriptional level. In 2011, Salmena et al. proposed the ceRNA hypothesis, as a supplement to the traditional miRNA-to-RNA theory, and demonstrated that some lncRNAs and circRNAs competitively bind miRNAs [31]. Many researchers have also confirmed, through well-designed experiments, that multiple circRNAs bind microRNAs through a ceRNA mechanism and consequently regulate target gene expression in PDAC, thus indicating that the ceRNA mechanism reasonably influences the occurrence and progression of pancreatic cancer [19, 32, 33]. In the present study, through bioinformatics analysis and luciferase assays, we determined that miR-451a is a target miRNA of mmu-cic-0001261. Moreover, we observed that the changes in the expression of mmu-cic-0001261 were opposite from those of miR-451a. Further rescue experiments indicated that mmu-cic-0001261’s promotion of cellular survival and neurite extension of DRG cells was facilitated by miR-451a inhibition. Therefore, mmu-cic-0001261 was found to competitively bind miR-451a and induce neuritogenesis in DRG cells.

**Fig. 8:**
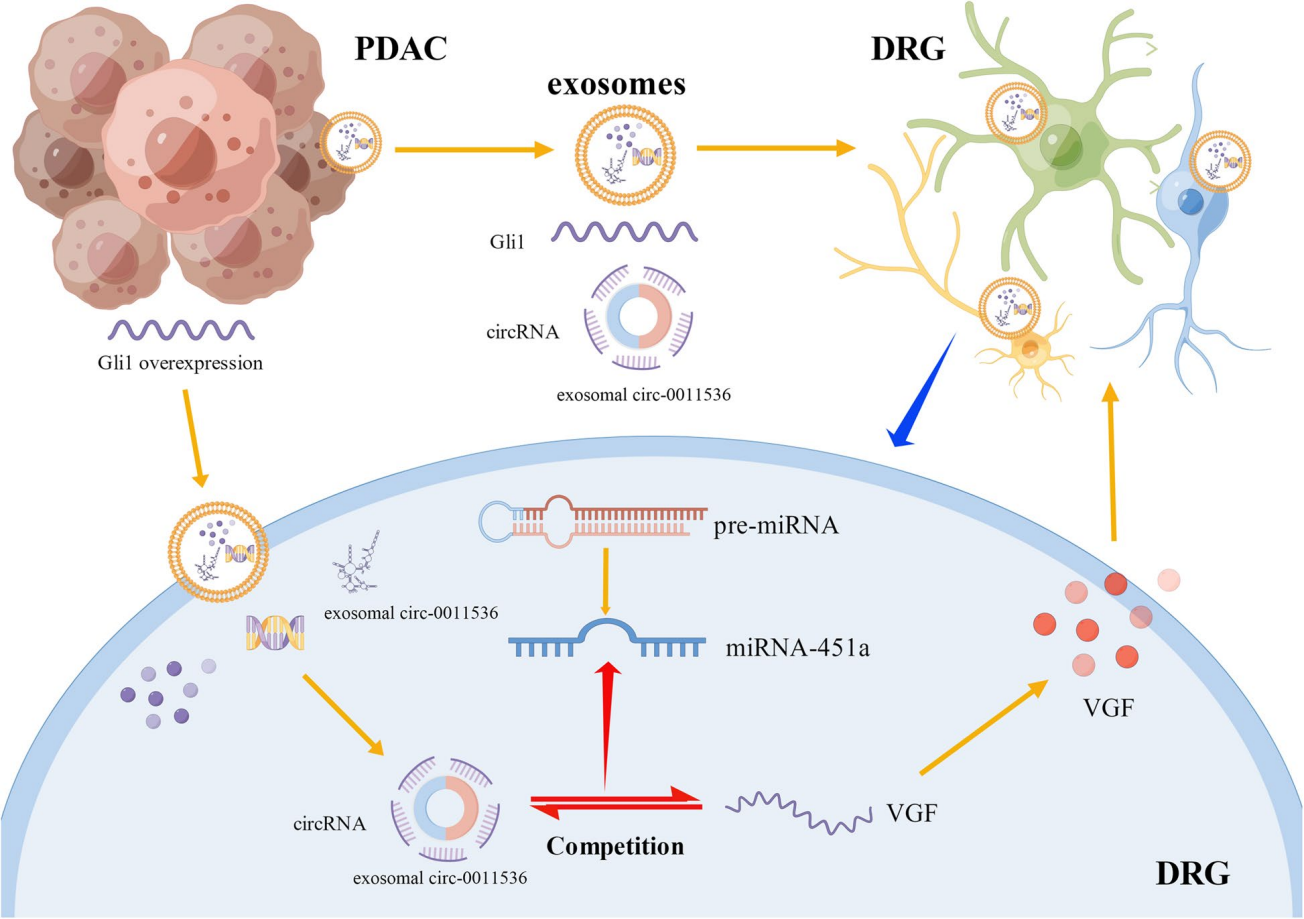
Action of PDAC-derived exosomes on DRG. PDAC of Gli1 overexpression-derived exosomes that were ingested by DRG cells. The has-circ-0011538 was transferred to DRG cells and competed with miR-451a for binding to VGF, which regulated the nerve alterations (figure created with Figdraw)

Canonically, microRNAs induce degradation or delay translation of target mRNA by binding the 3ʹ untranslated region. Here, through bioinformatics analysis, we showed that miR-451a may potentially target VGF, as further indicated by luciferase reporter assays. In addition, we demonstrated the suppressive effect of miR-451a on VGF expression in DRG cells. This effect was reversed by increasing mmu-cic-0001261 expression. VGF is abundant in both the peripheral and central nervous systems, and is also expressed in neuroendocrine cells from the gut [34–36]. Its expression is modulated by neurotrophins during axonal sprouting and synaptic remodeling, and it contributes to neuronal cell survival and plasticity [37–39]. Consistently, depletion of VGF abolished the mmu-cic-0001261-promoting effects of cellular survival and neurite extension in DRG cells in vitro. Together, our findings indicated that mmu-cic-0001261 modulates the neuroplasticity response of DRG cells by targeting the miR-451a/VGF axis. After identifying that mmu-cic-0001261 modulated nerve cell status in vitro, we provided further verification by establishing a plausible correlation between the presence of mmu-cic-0001261 and PNR in vivo. Notably, the expression level of mmu-cic-0001261 in the serum of lenti-Gli1 PDAC mice positively correlated with intra-PDAC nerve density, and has-circ-0011536 was found to be elevated in patients with PNR, thereby suggesting that has-circ-0011536 may be a valuable biomarker of nerve alterations in PDAC with activated hedgehog-Gli1 signaling (Fig. 8).

In summary, our study indicated that exosomal circ-0011536 modulates nerve alterations in PDAC through activated hedgehog-Gli1 signaling. Furthermore, our findings showed that circ-0011536 promotes VGF expression by sponging miR-451a. The unique advantage of the nuclease resistance of exosomes and circRNAs makes them excellent potential tools for research on new biomarkers and biological mechanisms. In the future, the signal transduction pathway between pancreatic cancer and nerve cells will require further study to establish a theoretical basis for ultimately finding a therapeutic strategy to block the neural invasion pathway.

### Supplementary Information

**Additional file 1.** Nucleotide sequences of primers used for qRT-PCR.

**Additional file 2: Supplementary Figure 1.** Gli1 expression by qrtPCR and western blotting in five different PDAC cell lines.

**Additional file 3: Supplementary Figure 2.** Representative PGP9.5 and S100B immunostaining in mice PDAC tissues.

## Data Availability

The datasets used or analyzed during the current study are available from the corresponding author on reasonable request.
