# Supplementary material for: Hedgehog-Gli1-derived exosomal circ-0011536 mediates peripheral neural remodeling in pancreatic cancer by modulating the miR-451a/VGF axis
Source: J Exp Clin Cancer Res. 2023 Dec 2;42:329. doi: 10.1186/s13046-023-02894-9 (PMC10693175; doi:10.1186/s13046-023-02894-9)
Supplement: Supplementary file 1 — Additional file 1. Nucleotide sequences of primers used for qRT-PCR. [file 13046_2023_2894_MOESM1_ESM.docx]

Nucleotide sequences of primers used for qRT-PCR.

| Species | Gene |  | Primer sequence (5’—3’) |
| --- | --- | --- | --- |
| Mouse | GFRA2 | Forward | CGCCTCTCGGACATCTTCAG |
|  |  | Reverse | TTGCAGTTGTCGTTCAGGTTG |
|  | IGSF21 | Forward | GCGGCTACCTGACAGTCAAC |
|  |  | Reverse | AACATGGCGTCAAACGTGAAG |
|  | VGF | Forward | AAGGATGACGGCGTACCAGA |
|  |  | Reverse | TGCCTGCAACAGTACCGAG |
|  | ROBO3 | Forward | AGATGAACTTGTTCGCGGACT |
|  |  | Reverse | GGAAGCAGACTAGGGTTGAGC |
|  | UNC5C | Forward | CTGCGGACTGGGACTAGGATA |
|  |  | Reverse | GGTTTCTGGGAGTTCGTGAAAA |
|  | NRG2 | Forward | GGAGGGCAAGGTACAGGGA |
|  |  | Reverse | AGGCTGTCTTAAAAACTAAGGGC |
|  | FGF14 | Forward | CCCCAGCTCAAGGGCATAG |
|  |  | Reverse | TGATGGGTAGAGGTAACCTTCTC |
|  | Gli1 | Forward | CCAAGCCAACTTTATGTCAGGG |
|  |  | Reverse | AGCCCGCTTCTTTGTTAATTTGA |
|  | circ-0001261 | Forward | TGTGATGAAGGCAAAATATACTGG |
|  |  | Reverse | CTCAAATAACGTCAAGGCAA |
|  | miR-451a | - | CACGCTAGACTGAGGCTCC |
|  | miR-423-5p | - | CTGGGAGAAGGCTGTTTACTCT |
|  | miR-6921-5p | - | GGGCACGGGACGCGGAAA |
|  | U6 | Forward | AAAGCAAATCATCGGACGACC |
|  |  | Reverse | GTACAACACATTGTTTCCTCGGA |
|  | GAPDH | Forward | AATGGATTTGGACGCATTGGT |
|  |  | Reverse | TTTGCACTGGTACGTGTTGAT |
| Human | Gli1 | Forward | AGCGTGAGCCTGAATCTGTG |
|  |  | Reverse | CAGCATGTACTGGGCTTTGAA |
|  | circ-0002487 | Forward | AAATCTCAGCGAGCAGTACC |
|  |  | Reverse | CATGCTGCTGGAATGAGACTG |
|  | circ-0004083 | Forward | TTGTCCTCCCCTTTCATCCCT |
|  |  | Reverse | GGATAAATAGCCAGCCCGAT |
|  | circ-0006646 | Forward | GTCCTCTACCAGCCATGCATC |
|  |  | Reverse | AAACAGTCATTCTCTTGCGGAT |
|  | circ-0011536 | Forward | CCACAGCAGGGACTACTAGACA |
|  |  | Reverse | AGATCCTGTTTGATTCGGCAT |
|  | circ-0003124 | Forward | GCCTCCTGGTTATTTGACCTT |
|  |  | Reverse | TGGCTTTGCTGGTATTATCACA |
|  | circ-0001524 | Forward | GTTCCGACGTTTCACAGTCA |
|  |  | Reverse | TCAGTTCCTGCTAGCTGTTCC |
|  | circ-0002696 | Forward | TCCTCCTACCAGCCATGCATC |
|  |  | Reverse | ATTTATTAGCAAAGGGGTCA |
|  | circ-0007229 | Forward | TGCCAGAGTTGTTTCTTTTCGG |
|  |  | Reverse | TCATTTTGGTTCAACTTGTGCT |
|  | circ-0084464 | Forward | GCGCGAATATCGTCATCGTT |
|  |  | Reverse | AGTATCCATGTAATCTCGGTCT |
|  | U6 | Forward | GGAACGATACAGAGAAGATTAGC |
|  |  | Reverse | TGGAACGCTTCACGAATTTGCG |
|  | GAPDH | Forward | TGTGGGCATCAATGGATTTGG |
|  |  | Reverse | ACACCATGTATTCCGGGTCAAT |

OE

hsa_circ_0011536 GTGGTGGTATCATGGATACAGAAATGTCTGAAGATATAGACCACAACTTAACTCCTACCCTTGACAGCATGTCTTATGGAATGCCGAATCAAACAGGATCTGAAAATTCATTGCTGGATGAAGATGATTATTTTTTGAACTCTGGGGATCTTGCAGGAATTCCAGTCGTTGGTAGTGACAATGAGGATGAACAGGATTTTAGTTCAAAGGACAATCTTGTTTCTTCAATTCATACTGATGATAGCTTGGAAGTAGAGAGAAGAGTCACACAGCATGAATCAGACAATGAAAATGAAATACAAATTCAAAATAAGTTAAAAAAAGACTTTCCTAAACAATTTGATCAGGTTTCTGTCTTTAAATCAATACGGAAAGATTTTAGTCTAGTAAGAGAAAACAGCAAAGAGACATTTTCTGGAAAGGAGAAAAATAGAGACCTAACTTATGAACGTGAAAAACGGTTGGATAAACCCCATAAAGATTTGGATTCAAGGTTGAAAAGCAGTTTTTTTGATAAAGCAGCTAATCAAGTTGAAGAAACATTACATACCCATTTACCACAAACCCCAGAAACAAACTTTAGGGATTCCAGCTACCCATTTGCCAATAAAGAATCCATTGGTTCGGAACTGGGGAATTCCTTTGCATCAAATATTAGAATTAAAGAAGAACCTTTGGATGATGAGTATGACAAAGCAATGGCACCACAGCAGGGACTACTAGACAAAATAAAAGATGAACCTGACAATGCTCAA

mmu_circ_0001261

GTGGTGTCATGGATACAGAAATGTCTGAAGATACAGACCACAACTTAACTCCCACCCTTGCCAGCATGTCTTATGGAATGCCGAATCAAACAGGATCTGAAAATTCATTGCTGGATGAAGATGATTATTTTTTGAACTCTGGGGATCTTGCAGGAATTCCAGTCGTTAGTAGTGACAATGAGGATGAACAGGATTGTAGTTCAAAGGACAACCTTGTTTCTTCAGTTCACACTGATGGTAGTTTGGAAGTAGAGAGAAGAGCTGCTCATCAGGAATCAGACAATGAAAATGAAATACAAATTCAAAATCAGTTAAAAAAAGACTTTCCTAAACAGTTTGATCAGGTTTCTGTCTTTAAATCAATACGAAAAGATTTTTGTTTAGTGAGAGAAAACAGCAAAGAGACATTTTCTGGAAAGGAGAAAAATAGAGACCTAACTTATCATGAACGTGAAAAACGGTTGGATAAACCCCATAAAGGTTTGGATTCAAGGTTGAAAAGCAGTTTTTTTGATAAAGCAGCTAATCAAGTTGAAGAAACATTACATACTCATTTACCACAAAACCCAGAAACAAACTTTAGGGACTCCAGCTACCCATTTGCCAGTAAAGAATCCATTGGTTCGGAACTGGGGAATTCGTTTGCATCAAATATTAGAATTAAAGAAGAACCTTTGGATGATGAGTATGACAGAGCAGTGGCGCCTCAGCAGGGACTACTAGACAGAGTTAAAGACGAACCTGACAATGCTCAA

siRNA

| si-1 sense | AUGCUCAAGUGGUGGUAUCAUTT |
| --- | --- |
| si-1 antisense | AUGAUACCACCACUUGAGCAUTT |
| si-2 sense | GACAAUGCUCAAGUGGUGGUATT |
| si-2 antisense | UACCACCACUUGAGCAUUGUCTT |
| si-3 sense | CUCAAGUGGUGGUAUCAUGGATT |
| si-3 antisense | UCCAUGAUACCACCACUUGAGTT |
