# Supplementary material for: Hedgehog-Gli1-derived exosomal circ-0011536 mediates peripheral neural remodeling in pancreatic cancer by modulating the miR-451a/VGF axis
Source: J Exp Clin Cancer Res. 2023 Dec 2;42:329. doi: 10.1186/s13046-023-02894-9 (PMC10693175; doi:10.1186/s13046-023-02894-9)
Supplement: Supplementary file 2 — Additional file 2: Supplementary Figure 1. Gli1 expression by qrtPCR and western blotting in five different PDAC cell lines. [file 13046_2023_2894_MOESM2_ESM.docx]

**Supplementary Figure 1.**

**
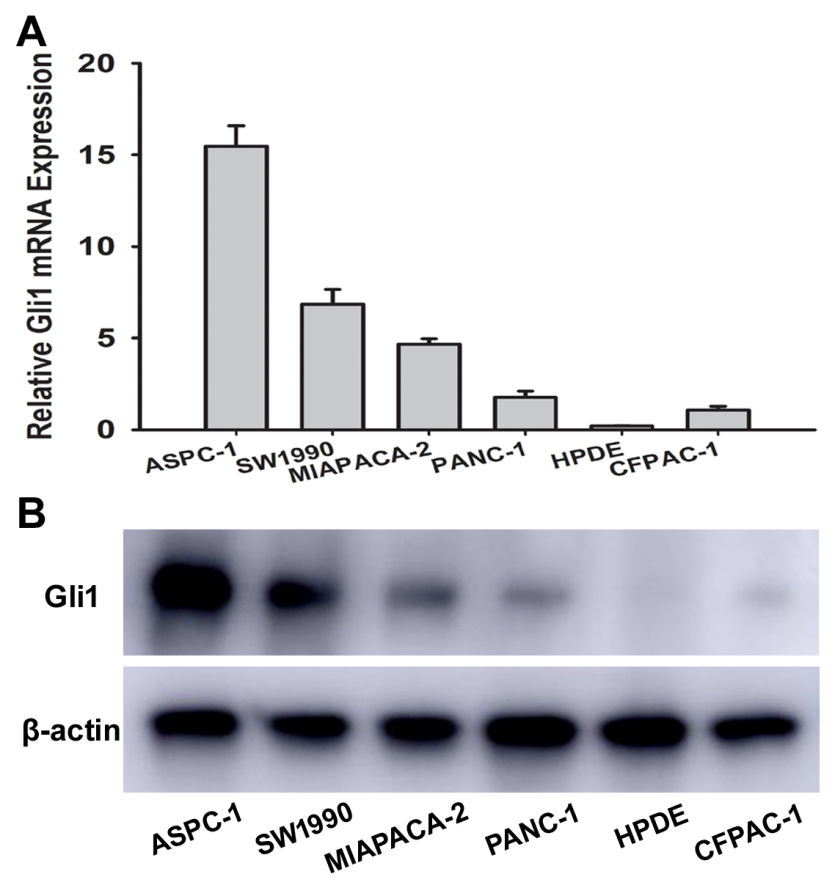
**

**Supplementary Figure 1.** **Gli1 expression by qrtPCR and western blotting in five different PDAC cell lines.** (A-B) qrtPCR (A) and western blot (B) analysis of Gli1 expression in total cell extracts from PDAC cells and normal pancreatic ductal epithelial cells (HPDE). β-actin was used as a loading control.
