# Supplementary material for: Hedgehog-Gli1-derived exosomal circ-0011536 mediates peripheral neural remodeling in pancreatic cancer by modulating the miR-451a/VGF axis
Source: J Exp Clin Cancer Res. 2023 Dec 2;42:329. doi: 10.1186/s13046-023-02894-9 (PMC10693175; doi:10.1186/s13046-023-02894-9)
Supplement: Supplementary file 3 — Additional file 3: Supplementary Figure 2. Representative PGP9.5 and S100B immunostaining in mice PDAC tissues. [file 13046_2023_2894_MOESM3_ESM.docx]

**Supplementary Figure 2.**

**
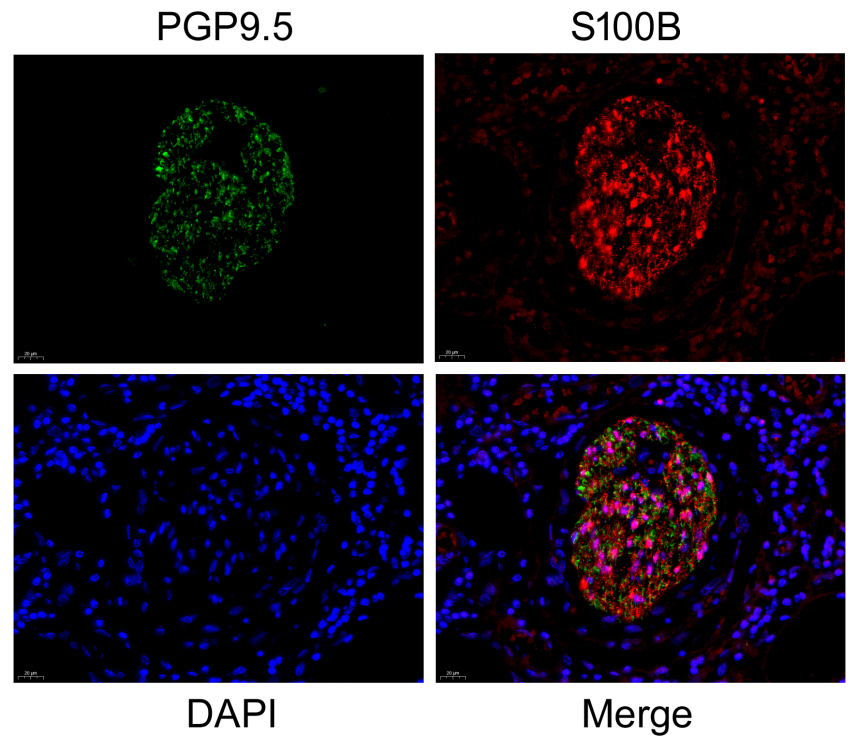
**

**Supplementary Figure 2. Representative PGP9.5 and S100B immunostaining in mice PDAC tissues.**
